# A chemogenomic approach to identify personalized therapy for patients with relapse or refractory acute myeloid leukemia: results of a prospective feasibility study

**DOI:** 10.1038/s41408-020-0330-5

**Published:** 2020-06-03

**Authors:** A. Collignon, M. A. Hospital, C. Montersino, F. Courtier, A. Charbonnier, C. Saillard, E. D’Incan, B. Mohty, A. Guille, J. Adelaïde, N. Carbuccia, S. Garnier, M. J. Mozziconacci, C. Zemmour, J. Pakradouni, A. Restouin, R. Castellano, M. Chaffanet, D. Birnbaum, Y. Collette, N. Vey

**Affiliations:** 1 Haematology Department, Institut Paoli-Calmettes, Aix-Marseille Université, Marseille, France; 2 Inserm, CNRS, Institut Paoli-Calmettes, CRCM, TrGET Preclinical Platform, Aix-Marseille Université, Marseille, France; 3 Inserm, CNRS, Institut Paoli-Calmettes, CRCM, Predictive Oncology, Aix-Marseille Université, Marseille, France; 4 Department of Biopathology, Institut Paoli-Calmettes, Marseille, France; 5 Department of Clinical Research & Innovation, Institut Paoli-Calmettes, Biostatistics & Methodology Unit, Aix Marseille Université, INSERM, IRD, SESSTIM, Marseille, France; 6 Department of Clinical Research & Innovation, Sponsor Unit, Institut Paoli-Calmettes, Marseille, France

**Keywords:** Acute myeloid leukaemia, Cancer genomics, Translational research

## Abstract

Targeted next-generation sequencing (tNGS) and ex vivo drug sensitivity/resistance profiling (DSRP) have laid foundations defining the functional genomic landscape of acute myeloid leukemia (AML) and premises of personalized medicine to guide treatment options for patients with aggressive and/or chemorefractory hematological malignancies. Here, we have assessed the feasibility of a tailored treatment strategy (TTS) guided by systematic parallel ex vivo DSRP and tNGS for patients with relapsed/refractory AML (number NCT02619071). A TTS issued by an institutional personalized committee could be achieved for 47/55 included patients (85%), 5 based on tNGS only, 6 on DSRP only, while 36 could be proposed on the basis of both, yielding more options and a better rationale. The TSS was available in <21 days for 28 patients (58.3%). On average, 3 to 4 potentially active drugs were selected per patient with only five patient samples being resistant to the entire drug panel. Seventeen patients received a TTS-guided treatment, resulting in four complete remissions, one partial remission, and five decreased peripheral blast counts. Our results show that chemogenomic combining tNGS with DSRP to determine a TTS is a promising approach to propose patient-specific treatment options within 21 days.

## Introduction

Treatment of acute myeloid leukemia (AML) has improved over the past 30 years. Initially, progress was due to the optimization of the “one-size-fits-all” chemotherapy-based approach. Recently, several targeted agents have emerged to change the treatment landscape of AML. In spite of these improvements, the majority of AML patients relapse and succumb to disease with a 5-year overall survival rate of around 20%^1^. Clearly, new therapeutic strategies are required for patients with refractory or relapsed disease. Among them, personalized treatment approaches are appealing but have yet to be extensively studied in this setting. The development of genome sequencing has allowed a better understanding of the mechanisms of leukemogenesis by identifying numerous somatic genetic alterations^2,3^, revealing the molecular AML heterogeneity. Some of these alterations, called “actionable mutations”^4^, lead to cancer cell vulnerabilities that could be targeted by specific drugs to improve the outcomes of patients. The success of targeting the Breakpoint Cluster Region-Abelson fusion protein fusion with imatinib to control chronic myeloid leukemia (CML)^5,6^ has promoted the development of precision medicine. However, it remains challenging to assess the potential of actionable mutations for successful targeting and disease control in AML because of the increased genomic complexity compared to CML^4,7^. Moreover, genomic data alone provide limited value in developing precision medicine with some studies showing improvement of overall response rate and/or progression-free survival (PFS) using matched targeted therapies^8–10^ and others not^11^. Another limitation to the development of precision medicine is that relatively few agents exist to target actionable genes (*PML-RARA, FLT3, KIT, IDH1*, and *IDH2)*. Furthermore, the response to targeted therapies used as monotherapies are generally short-lived, mainly due to the development of drug resistance by the tumor^12^, necessitating the targeting of different pathways by using drug combinations^13,14^ while minimizing toxicities^15^. Thus, functional approaches such as ex vivo drug sensitivity and resistance profiling (DSRP) may complement genomic data to identify new targeted therapies and augment the clinical toolbox with existing drugs that can be tailored to larger numbers of patients with AML^16^. This process can test simultaneously dozens of drugs ex vivo in a rigorous concentration response format, enabling the identification of a drug response profile for each patient^17–21^ independent of genomic profiling. The addition of genomic analysis to DSRP gives rise to a more powerful analysis called “chemogenomics” that has the potential to identify more effective treatment options for a given patient, as well as discover unexpected correlations between molecular profiles and drug response to uncover new indications (drug repositioning) or reveal new mechanisms of action. Following the initial report by Pemovska et al.^18^, several studies have confirmed the potential of this approach^19,21^. However, its feasibility has not been evaluated in the clinical context of a potentially rapidly progressive disease such as refractory/relapsed AML. We thus designed a prospective study that serves as a clinical proof of concept for a chemogenomic approach demonstrating its ability to produce a treatment-tailored strategy (TTS).

## Patients and methods

### Study design

The primary objective of the CEGAL-IPC-2014-012 study was to determine the proportion of eligible patients for whom the results of chemogenomic analysis could be obtained in <21 days after blood and bone marrow sampling. To be eligible for this prospective single center study, patients had to be age >18 years, have non-promyelocytic AML according to WHO criteria^22^, be refractory to- or have relapsed after at least one line of prior conventional chemotherapy or hypomethylating agent therapy, have an ECOG performance status (PS) < 3 and an estimated life expectancy >3 months. Written consent was obtained from all patients. The trial was approved by the Ethics Committee in accordance with local policy and registered in clinicaltrials.gov (NCT02619071).

Molecular/genetic profiling (see Supplemental Material and Methods and Table S1)

Ex vivo drug sensitivity and resistance profiling (DSRP) (see Supplemental Material and Methods and Table S2)

### Drug choice and treatment-tailored strategy (TTS)

To further identify drugs with a patient-specific efficacy, we used a *Z-*score (defined as: patient EC50–meanEC50 of a patient reference matrix/standard deviation, in which the reference matrix was previously defined on a panel of 25 different samples treated identically).This *Z-*score permits objective identification of a patient cell sensitivity using a quantitative threshold. In practice, a lower *Z-*score indicates greater sensitivity of the patient’s cells as compared to those of the other patients.

As soon as the genomic and DSRP data were available, a multidisciplinary review board (MRB) was organized with physicians and molecular biologists to discuss the results. To choose the most efficient drugs for a given patient, we first selected all the drugs with a *Z-*score < −0.5 (arbitrarily set threshold) then narrowed to the 5 most potent drugs related to a detected actionable mutation. For some patients having no drugs with a *Z-*score < −0.5, we could still propose drug compounds showing activity comparable to that measured on the samples in the reference matrix and offering the best difference with the activity measured on peripheral blood mononuclear cells (PBMCs).

Then, a tailored treatment strategy-TTS-(mono or polytherapy) was proposed based on the identification of actionable mutations and/or potential related drug responses. For choosing combinations when several drugs were proposed, we took into account: the accessibility of the drugs in a reasonable timing, the potential toxicity of the combination or the knowledge of an already used combination in the literature data.

The physician was then free to follow or not the TTS.

### Statistical analysis

The rate of patients for whom chemogenomic analyses were available in <21 days was estimated, with its Wald’s bilateral 90% confidence interval. The main objective was to demonstrate that this rate is significantly superior to 30% (unacceptable rate). For this purpose, a right-sided *Z*-test for comparison to the theoretical value of 30% was performed at the significance level *α* = 0.05.

For the global analyses and inter-patient EC50 comparison, we normalized all the EC50s of one drug with the EC50max of this drug obtained in the cohort. Thus, all the EC50s were comprised between 0 (sensitive) and 1 (resistant).

Association tests between mutations and EC50 were done with a *t*-test and *p*-values and were corrected for multiple testing with the Benjamini–Hochberg method. Of the 232 variables associated with the mutations and 152 drugs (76 drugs in bone marrow +76 in blood, 35,264 possible tests), only the variables with *n* ≥ 4 mutations and with one EC50 measurement were kept. Moreover, if the samples had a standard deviation of EC50 < 0.05 for a given drug, the drug was not tested.

Overall survival was calculated using the Kaplan–Meier method from the date of inclusion in the trial to the date of death or last follow-up, whichever came first.

## Results

### Patient characteristics

#### Clinical and biological characteristics

A total of 55 patients were included between August 2015 and August 2018. Their characteristics are listed in Table 1. Median age was 65 years (range 24–81) and median ECOG-PS was 1. The majority of patients had high-risk disease according to European Leukemia Net (ELN) 2017 classification (*n* = 36, 65%) and had treatment-refractory disease (*n* = 46, 84%). Median number of *white blood cells* (WBC) was 3.6 G/L (1.1–51.3), with four patients (3%) having WBC above 20 G/L. Forty percent of the patients had received more than three lines of treatment and the mean number of prior therapies was 2.2 (range 1–5), including 12 patients (22%) who previously underwent allogeneic stem cell transplantation.

**Table 1:** Patients’ characteristics at inclusion.

Characteristics at inclusion *n* = 55	
Median age (yo, range)	65 (24–81)
Sex M/F (number, %)	
Male	33 (40)
Female	22 (60)
Performance status (number, %)	
0	8 (14)
1	35 (64)
2	12 (22)
Status at inclusion (number, %)	
Relapse	9 (16)
Refractory	46 (84)
Number of prior therapies (number, %)	
1	16 (29)
2	17 (31)
≥3	22 (40)
Median number of WBC G/L (range)	3.6 (1.1–51.3)
Median number of medullary blasts % (range)	47.5 (1–95)
WHO classification (number, %)	
inv(16)(p13.1q22)	2 (4)
inv(3)(q21.3q26.2)	3 (5)
t(6;9)(p23;q34.1)	2 (4)
Mutated NPM1	5 (9)
MRC	32 (60)
Therapy related	4 (7)
NOS	5 (9)
CMML-2	2 (4)
ELN classification (number, %)	
Adverse	36 (65)
Intermediate	15 (27)
Favorable	4 (7)

#### Genomic characteristics

**Fig. 1:**
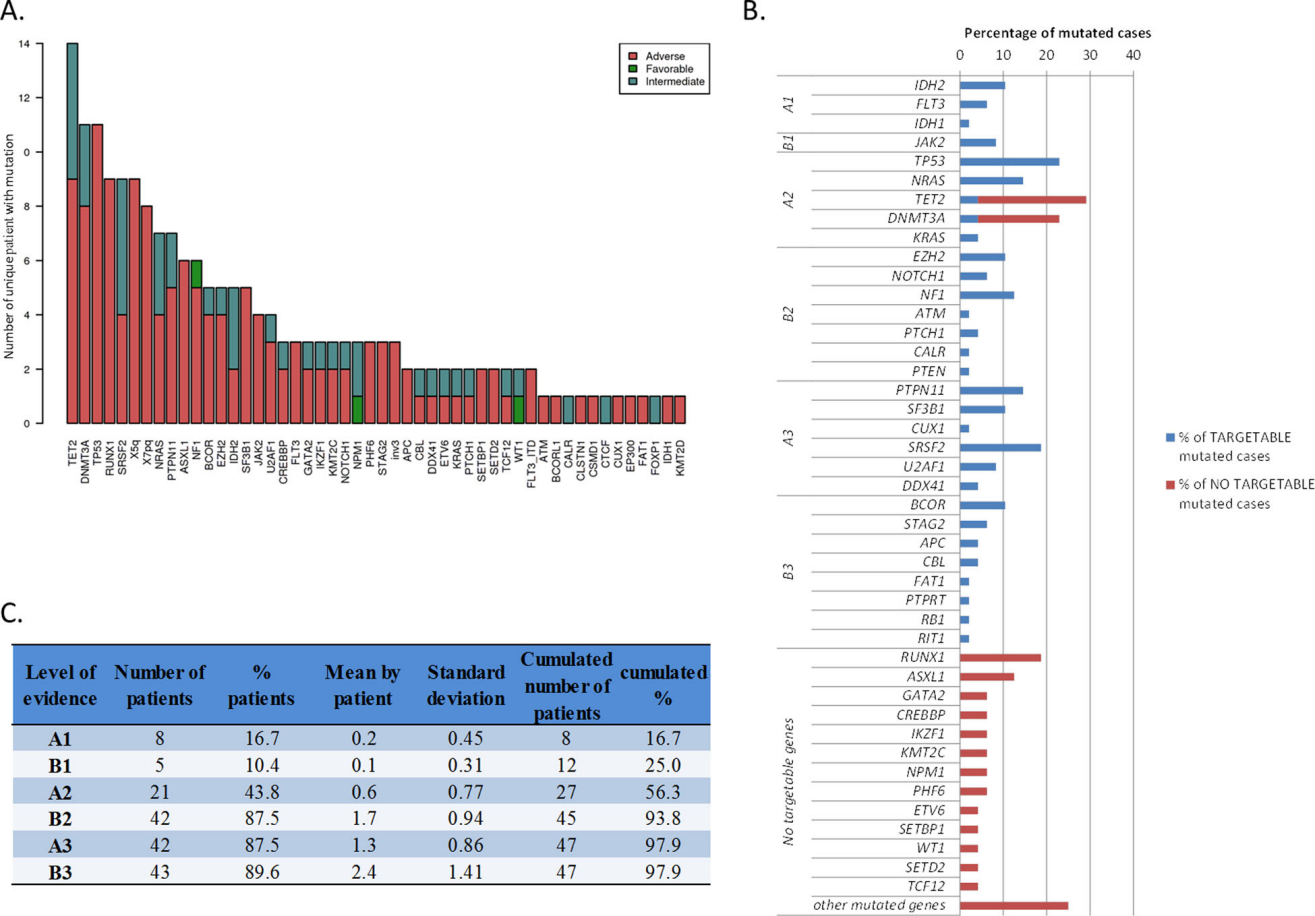
Targeted Next Generation Sequencing (tNGS) analysis results. **a** Genomic distribution of all the mutations found in the CEGAL cohort with the corresponding ELN classification. **b** Representation of the different actionable mutations found in the cohort according to the algorithm (23) regardless of their level of evidence. **c** Distribution of the mutations according to their level of evidence in the scientific literature.

We identified mutations in 63 genes with at least one mutation per patient (Fig. 1a). In our heavily pretreated population, the median number of mutated genes and mutations per patient was 3.8 (range 1–10) and 4.2 (range 1–11), respectively. Eight patients (17%) harbored >6 mutations. The most frequent mutations were in *TET2* (29%), *DNMT3A* (*23%*), *TP53* (23%), *RUNX1* (19%), and *SRSF2* (19%). *FLT3* and *NPM1* mutations were found in 3 patients each (6%). The most frequently altered classes of genes were signaling pathway (in 54% of the patients), and chromatin modifiers (54%), DNA methylation (48%) and transcription factors (40%) (Fig. S1).

### TTS design

#### Actionable mutations

We found potential actionable mutations (as described above) in at least 16 genes and among 42 patients (94%) (Fig. 1b, c). Also, we found that 17% of the patients had an A1 alteration *(FLT3*, *IDH1*, *IDH2*), 10.4% a B1 alteration, 44% an A2 alteration (*TET2*, *TP53*, *NRAS*, *KRAS*, *JAK2*, *DNMT3A)* and 88% a B2 alteration (Fig. 1c). The most frequently mutated actionable genes were *TP53* (11 patients), *NRAS* (7 patients), *NF1* (6 patients), and *IDH2* (5 patients).

#### Drug sensitivity and resistance profiles

Among the 32 patients for whom blood and bone marrow samples were analyzed, EC50 were comparable for the two samples, except for three of them (difference not explained by a difference in blast percentage between blood and bone marrow) (Fig. S2), indicating that each of leukemic cells source could be used.

**Fig. 2:**
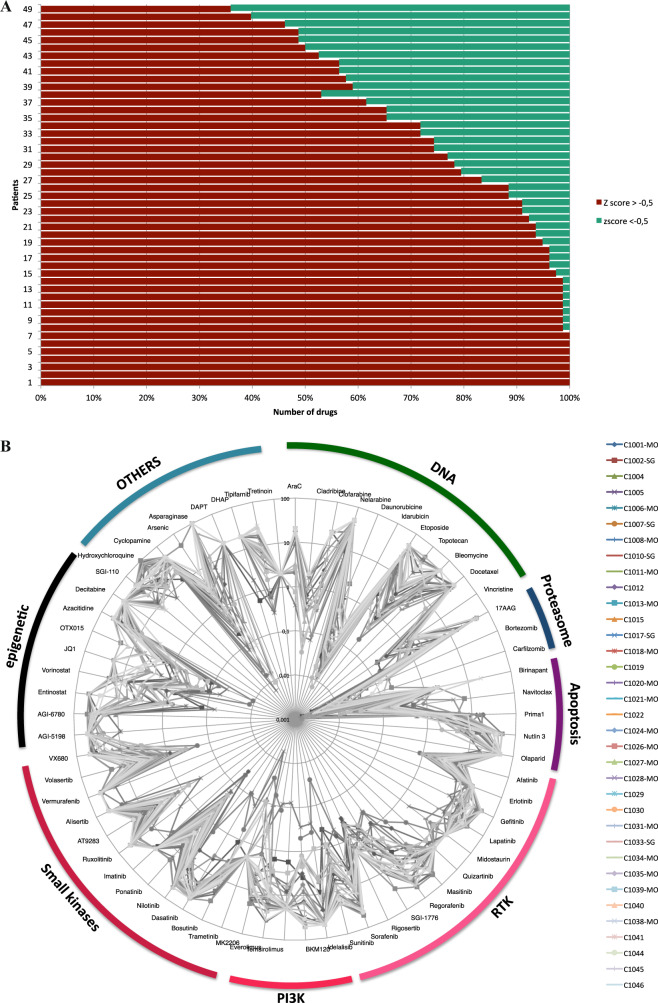
Drug Sensitivity Resistance Profiling (DSRP) results. **a** Barplot representing the percentage of drugs with a *Z-*score<−0.5 for a given sample. **b** Representation of the individual drugs’response of all the patients with each line representing one patient and each point the EC50 value of the correspondent drug (logarithmic scale, the lowest EC50 in the center).

A high variability in drug response was observed across all samples in terms of EC50 and *Z-*score. The number of effective drug candidates (with a *Z*-score < −0.5) varied from 0 to 53 according to the patients (Fig. 2a). Forty-one patients (85%) had at least one drug with a *Z-*score < −0.5. The median number of drugs with *Z*-score < −0.5 was 9. For two patients who had not drugs with a *Z-*score < −0.5, we selected one drug with a *Z-*score > −0.5 because the EC50 was low and the drug matched to an actionable mutation. Five patients (10%) were considered as resistant to the entire drug panel.

We observed for example that bortezomib and carlfizomib had a low EC50 in a majority of samples (Fig. 2b). In contrast, the IDH inhibitors (AGI 5798 and 6780) or decitabine and to a lower extent azacitidine were not active in most of the samples. The response to certain drugs such as asparaginase or PI3K inhibitors showed a strong variation from one patient to another.

Among the 78 drugs of the panel, 50 were selected by the board meeting for at least one patient. An average of 3.4 potentially active drugs per patient was proposed. The most frequent drug classes based on DSRP results were *kinase inhibitors* (52 times), *chemotherapies* (36 times), *epigenetic drugs* (32 times), and *apoptosis inducers* (25 times). The most often selected compounds were tyrosine kinase and PI3K inhibitors (as *Kinase inhibitors*), clofarabine and aracytine (as *chemotherapies*), hypomethylating agents especially azacitidine and SGI 110, BRDi (as *epigenetic drugs*) and PRIMA1 (as *apoptosis inducer*) (Fig. S3).

#### Feasibility of the treatment-tailored strategy

**Fig. 3:**
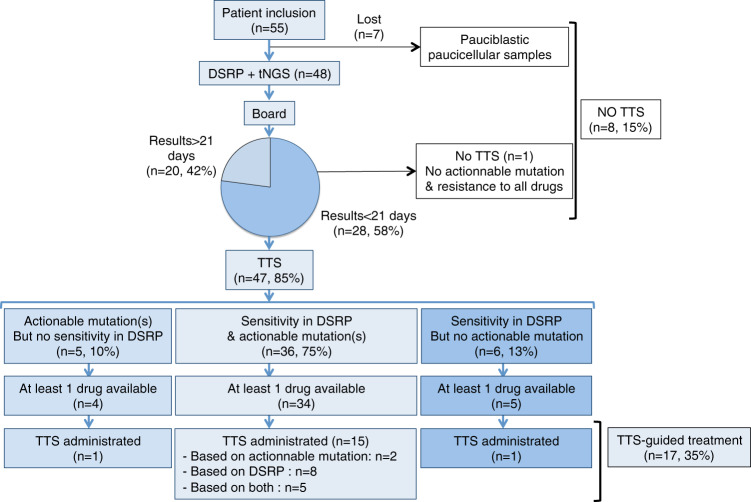
Flow chart summarizing the results of the chemogenomic approach applied to the CEGAL cohort. DSRP Drug Sensitivty Resistance Profiling, tNGS targeted Next Generation Sequencing, TTS Tailored Treatment Strategy.

Median times from sampling to DSRP or to targeted next-generation sequencing (tNGS) analysis were, respectively, 6 days (range 3–14) and 15 days (range 7–37). Median time for TTS proposal by the MRB was 18.5 days (range 8–50). The main objective was reached with chemogenomic analyses available in <21 days for 28/48 patients (58.3%, IC90% [46.6%–70.0%]) (Fig. 3). This rate was significantly superior to the objective of 30% (*p* < 0.0001, *Z*-test). Notably, 12 patients were discussed at day 22 and 23 after bone marrow samples.

Among the 55 patients included, a TTS could not be designed for eight patients (15%): for seven patients because the chemogenomic analysis could not be done due to an insufficient number of cells in the collected samples and for one additional patient because there were no actionable mutations and no sensitivity to any of the drugs included in the panel.

A TTS could be designed for 47 patients (85%) based on tNGS and/or DSRP results: tNGS only for five patients (10%), DSRP only for six patients (13%), and both tNGS and DSRP for 36 patients (75%). For the TTS, at least one of the selected drugs was available (commercially, off-label or in clinical trials) in 93% of the cases.

**Fig. 4:**
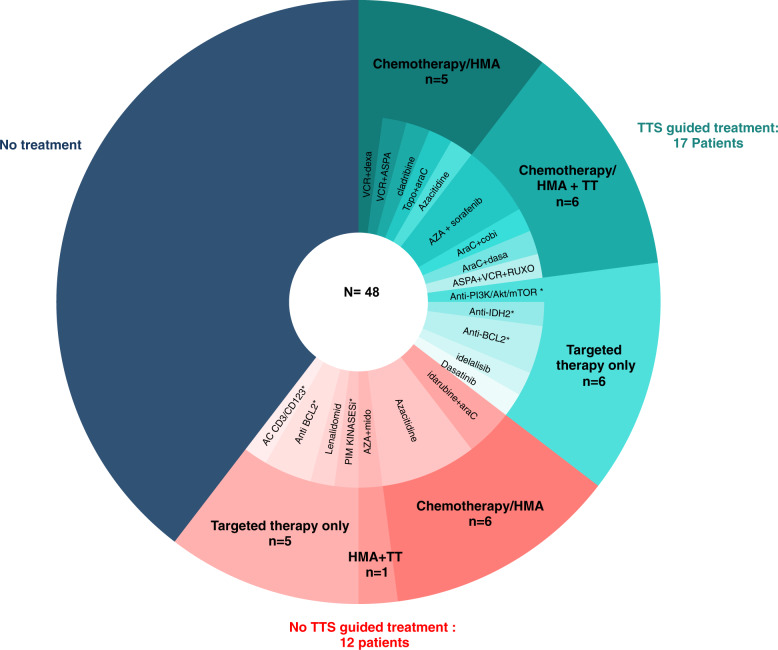
Treatment received by the patients after Multidisciplinary Review Board (MRB). TTS treatment-tailored strategy, TT targeted therapy, HMA hypomethylating agents, VCR vincristine, dexa dexamethasone, ASPA asparaginase, TOPO topotecan, araC aracytine, AZA azacitidine, soraf sorafenib, cobi cobimetinib, dasa dasatinib, RUXO ruxolitinib, mido midostaurine, AC antibody. Asterisk indicates molecule delivered in the context of a clinical trial.

Seventeen patients (31%) were treated according to the TTS (TTS group, Table S3); nine based on the DSRP results, three based on the presence of actionable mutations, and five based on having both (correlation between tNGS and DSRP). Personalized treatments consisted of targeted drugs in 12 patients (six monotherapy; six in combination) cytotoxic drugs in four patients and hypomethylating agent in one patient (Fig. 4).

Twelve patients were treated with another treatment than those recommended by the MRB (non-TTS group: inclusion in clinical trial for four patients, other treatment chosen by the physician for eight patients, no recommended available drug on market in four patients) and 19 patients did not receive any treatment or only palliative care (because of a poor general status or death before MRB).

#### Response and survival

In the 17 TTS patients, there were 4 complete response (CR) (two still alive in CR), one partial response, and five decreased peripheral blast counts. The seven remaining patients progressed. The four CR responses were obtained after allogenic stem cell transplantation with cladribine for one patient, after azacitidine + sorafenib for two patients and after azacitidine alone for the last one.

In the non-TTS group, there was only one partial response after azacitidine and no response in the palliative group.

This difference was not explained by any difference in median age (66 vs. 62 years for TTS group and non-TTS group, respectively, *p* = 0.5), nor median PS at inclusion (one in both group, *p* = 0.6) or median number of previous line (2.2 vs. 2.3, *p* = 0.7).

Median OS from inclusion was 3.3 months (0.2–32.3) in the whole cohort and was 5.6 (range 1.6–30.2), 6 (range 0.8–16.8), and 2 months (range 0.2–9.1) for the TTS group, the non-TTS group and the palliative group, respectively.

### Global analysis of drug sensitivity profiles

Using the median of all the normalized EC50s of the drug panel for each patient sample, we could evaluate the global sensitivity of each patient. Two groups of different sensitivity could be identified: one with high medians representing resistant patients and the other with middle or low medians representing the sensitive group illustrating that even heavily pretreated patients could still respond to therapy, at least in ex vivo (Fig. S4).

These two groups also appeared in the hierarchical clustering (Fig. S5). However, no correlation between these two groups and the karyotype, the ELN status, or the mutational status of one particular gene could be observed. Enrichment in mutated signaling genes was however noticed in the sensitive group and samples with at least one mutation in the signaling pathway class had a significantly lower median EC50 (*p* = 0.02, Fig. S6). More mutations (average 4 vs. 3) were observed in the resistant group as compared to the sensitive group but no difference in the number of previous line of therapy was observed. Although not significant, patients with secondary AML were globally more resistant than those with de novo AML (median normalized EC50 of patients with secondary AML were 0.83 vs. 0.47). There was no correlation between median EC50s and the median overall survival (*p* = 0.2). Samples with blasts <25% were associated with lower drug sensitivity (higher median EC50, *p* = 0.015, Fig. S7) and distributed mainly in the resistant group in the clustering.

Using the median of all normalized EC50s of every patient’s sample for each drug, we could compare the global activity level of each drug. The ten drugs with the highest activity, that is to say with the lowest median EC50s, were arsenic, daunorubicine, carfilzomib, bortezomib, entinostat, metformin, birinapant, JQ1, and ponatinib (Fig. S8). As described above, some drugs, especially asparaginase, had a high median that was skewed by high activity in a small number of samples. We then compared this median to the EC50 obtained for the same drug in PBMC (from eight healthy donors) to define which drugs were most specific towards leukemic cell. We observed that some drugs had a lower median EC50 on AML cells as compared to PBMCs, indicating that they were more leukemia specific (JQ1, 17AAG and entinostat are the first three, Fig. S9). In contrast, the average sensitivity to conventional chemotherapies did not differ between the patient samples and PBMC reflecting the known limited therapeutic window for these drugs and their lack of specificity.

In the clustering analysis (Fig. S5), we also observed that some drugs formed consistent clusters (cytarabine, cladribine, clofarabine, bleomycine, etoposide, idarubicine or also dasatinib, alisertib, imatinib, ruxolutinib, vermurafenib, trametinib), while others seemed less expected (hydroxychroroquine, bosutinib, ponatinib and volasertib).

### Correlation analyses between drug sensitivity and genomic profiles

Since our review board assessed actionable genetic alterations as defined by Perera-Bel et al.^23,24^, we first evaluated correlations between DSRP results and the actionable mutations found in the cohort (Table S4). Among patient samples exhibiting such an actionable alteration, 21 (50%) were sensitive to the associated drug. Yet not statistically supported, the most frequent correlations were sensitivity to P53 reactivator in TP53 mutated patients (7/11 patients), FLT3 inhibitors in *FLT3-*mutated patients (2/3 patients), to MEK inhibitors in patients with *RAS* mutations or with supposed activation of the RAS pathway (*PTEN* or *PTPN11* mutations, *NF1 loss*) or sensitivity to PI3K/AKT/MTOR inhibitors in patients with supposed activation of this pathway (for example, loss of *CUX1*). There were also discrepancies, notably for samples with *IDH2* mutations that were all resistant to the IDH2 inhibitor.

Although the limited number of patient makes the interpretation difficult, we next sought to evaluate whether unexpected mutation/drug pairs could be identified using our chemogenomic data. We tested 1679 mutation/drug associations, keeping all the associations with a fold change <0.2 and a false-discovery rate (FDR) < 0.05 (arbitrarily defined). We found 52 significant associations involving nine genes: *NRAS* (*n* = 27 associations), *TP53* (*n* = 6), *RUNX1* (*n* = 4), *JAK2* (*n* = 3), *EZH2* (*n* = 3), *PTPN11* (*n* = 3), *NF1* (*n* = 3)*, SF3B1* (*n* = 2), and *DNMT3A* (*n* = 1) (Fig. S10 and Table S5). The most significant associations were: (i) *NRAS* mutations associated with sensitivity to AT9283 (JAK 2/3, Aurora A/B, ABL^T315I^inhibitor, FDR = 2 × 10^6^), MK206 (Pan-AKT inhibitor, FDR = 2.64 × 10^5^), and VX680 (Aurora inhibitor, FDR = 5.82 × 10^5^) and (ii) *RUNX1* mutations with bleomycine (FDR = 2.64 × 10^5^). None of them were listed in the databases used by Perera-Bel et al.^25,26^, except for the association between *NF1* and sensitivity to BET inhibitors (B3, *p* = 0.02), *NRAS* and sensitivity to HSP90 inhibitors (B3, *p* = 0.01) and *TP53* and resistance to MDM2 inhibitors (A3, *p* = 0.01).

## Discussion

To our knowledge, this study of 55 AML relapse/refractory patients is the largest cohort studying the feasibility of a treatment-tailored strategy using a chemogenomic approach. Our main objective (availability of the results in 21 days) was reached for a majority of the patients (58%). This timing is compatible with the management of such patients whom survey rarely exceeds 6 months^27^. Based on the results of tNGS and/or DSRP, we were able to propose a TTS to 85% of the patients and eventually 30% received a personalized treatment. The main reasons for not receiving a personalized treatment were the general status of the patient, the choice of another therapy or the unavailability of the drug.

Although complete responses occurred in the TTS group, we were not able to show improvement in survival with personalized therapy but the number of patients was too small to highlight any difference and the cohort was composed of heavily pretreated patients with poor prognosis. The short overall survival of 3.3 months in the whole cohort is an argument for including patients earlier in their therapeutic course, for instance at first relapse.

In terms of genomics, our cohort had a distinctive mutational profile with a predominance of *TET2, DNMT3A*, and *TP53* mutations, which differs from the de novo leukemia profile^2^ and we show that 94% of the patients had actionable mutations. However, even if a patient harbors an actionable mutation, it does not predict response to the associated drug^28^ and actually a correlation between the actionable target and its matched drug was not always observed. There could be several explanations to the later.

First, all actionable mutations found in this cohort are not clinically validated and do not have the same scientific knowledge level. Since the development of NGS, some authors have tried to ease the classification of these actionable events^29^ and to generate tools to guide physicians in personalized treatment decisions^30^. We chose to classify the alterations by using the classification published in 2018, adding new actionable targets found in the literature such as *TET2*^31^. We found that 94% of the patients had at least a B2 alteration. Eventually, we found that 17% of the patients harbored an A1 actionable mutation for which a targeted treatment is approved in clinical practice, and 44% harbored an A2 actionable mutation for which drugs have been studied in clinical trials. We initially decided not to take A3/B3 level alterations in consideration because of the lack of validated data, but this might be reconsidered in view of the two significant A3 alteration/drug associations identified in our study. Second, the absence of efficiency of a drug on its paired target could be explained by the clonal heterogeneity and the predominance of some mutations within a clone. Currently, a clinical trial is ongoing to assess whether targeting a clonally dominant driver mutation improves outcome compared with targeting the same event in a subclone (DARWIN1, NCT02183883). Unfortunately, because of the small number of patients,only the mutated status, not the VAF, could be taken into account to test the correlation with the drug’s response. It will therefore be interesting to be able to increment our cohort to investigate these points and also to look at data from other cohorts (such as BEAT AML) in order to have a sufficient number of samples.

Eventually, in our cohort, genomic data guided a targeted treatment by its own in 8/48 patients (17%).

This is in favor of the use of additional tests to help clinicians in selecting the most appropriate therapy^16^. We chose DSRP as a functional test, because it may rapidly identify ex vivo potential (in vivo) efficient drugs and has already been used in several studies in AML^14,19,21,32^. In our study, the drug category that stood out as the most efficient and was the most often proposed in MRB was kinase inhibitors, which is consistent with the fact that signaling pathway alteration is a major mechanism in leukemogenesis. Conventional chemotherapy is also interesting because despite the refractory and multi-treated hallmark of the patients of our cohort, there still persists for many cases some sensitivity to chemotherapies that could find their place in association to other targeted treatments or in sequential conditioning regimens before allograft. Interestingly, proteasome inhibitors seemed to be efficient in a majority of the patients but we do not have a clear explanation and the search for possible correlations with genomic data has not so far provided us with any encouraging leads.

Finally, epigenetic drugs were also central in this cohort enriched in secondary AMLs with many mutations in genes encoding for chromatin modifiers and DNA methylation modulators. Response to drugs was variable and we identified two groups with different sensitivity profiles, one resistant and the other rather sensitive. Unfortunately, we were not able to identify any specific hallmark linked to these two groups, maybe because of the limited number of patients. The clustering was also informative about the mechanism of action of the drugs. Interestingly, vincristine clustered with rigosertib, which was recently described as a microtubule poison in addition to being a kinase inhibitor^33^ indicating that the observed clustering might reflect common targeted pathways. This might be in line with the study by Lin et al.^25^, which recently highlighted the off-target activity of various compounds in clinical development whose anti-proliferative efficacy was not affected by the loss of the target. Interestingly, the drug tests were reproducible between blood and bone marrow samples (except for three patients), which encourages us to do the future tests only on blood cells to avoid invasive bone marrow aspirates and save time for the analyses.

Eventually, the DSRP was informative in 41/48 patients (87%) with the identification of drugs predicted efficient, and led on its own to a personalized treatment in 14/48 patients (29% of the total cohort).

The fact that some targeted therapies were not efficient on their predicted molecular target (for example IDH2) could have several explanations. The first one, as said above, could be that the mutation targeted by the drug was in a minor clone, the counter selection of which had no impact on the disease. Unfortunately, we could not take into account the VAF of the mutation in the interpretation of the correlation tests. Second, the problem could come from the DSRP technique, which presents several pitfalls. Indeed, one of the biases could be the short (48 h) incubation time that could underestimate some drug effects. Indeed, drugs such as hypomethylating or differentiating agents need successive cell division cycles to reach substantial cellular impact. Moreover, with DSRP, we only focused on cellular viability/proliferation, whereas differentiation marker analysis by flow cytometry could be more relevant to evaluate the impact of drugs such as IDH2 inhibitors. Also, our DSRP methodology does not take into account the impact of the microenvironment known to increase chemoresistance and this indeed could affect ex vivo response^26^.

The absence of an objective cut-off to estimate ex vivo drug sensitivity remains a limitation towards the translation of DSRP results into clinical practice. Other studies have developed a drug sensitivity scoring (DSS) to evaluate drug potency for each patient^19,20^, but we chose instead to use a *Z*-score more classically used in high-throughput screening because it permits to identify patient’s specific vulnerabilities^34–36^. One of the evolutions of our approach has been to take into account the response of drugs with PBMC using the patient samples/PBMCsEC50’s ratio, which makes it possible to identify cancer-selective drugs with potentially less systemic toxicity. Moreover, data analysis indicated that samples with blasts <20% were resistant to almost all the drugs. The analysis of these patients does not seem to be relevant, and were thus excluded. Clearly, a common evaluation of these various parameters between several investigator centers will be necessary in the future in order to better define and refine the applications of these methodologies in optimized clinical practice.

An important future contribution to the evolution of the chemogenomic approach will be the ability to evaluate drugs combinations. As systematic testing of targeted agents with cytotoxic treatments or other targeted agents poses significant experimental challenges^37^, this should be effectively evaluated in the future. The principal limitation for this at the time of our study was the number of cells for each patient, which must be significant in case of combinations. Although possible, drug combination analysis remains, as our methodology on fresh samples is, accessible for a few pre-defined combinations and for biological samples with sufficient richness in cellular material. However, the test can be miniaturized at least to a 384-well plate format and an integrated use of functional drug screening combined with genomic and molecular profiling has recently been described to enable patient-customized prediction and testing of drug combination synergies for T-cell prolymphocytic leukemia patients^38^.

In conclusion, chemogenomic is an interesting and innovative approach feasible in a hospital setting and in a time frame adapted to refractory/relapsed AML patients. Further efforts should be devoted to the development of additional functional tests for the study of not so heavily treated patients. Whether this approach brings a real clinical benefit to patient is still not certain and must be studied in randomized controlled clinical trials.

## Supplementary information

Supplemental material
