# Supplementary material for: A chemogenomic approach to identify personalized therapy for patients with relapse or refractory acute myeloid leukemia: results of a prospective feasibility study
Source: Blood Cancer J. 2020 Jun 3;10(6):64. doi: 10.1038/s41408-020-0330-5 (PMC7266815; doi:10.1038/s41408-020-0330-5)

Supplemental Material and Methods

Molecular/genetic profiling

To identify clinically-actionable gene mutations and putative druggable pathways, genomic copy number aberrations (CNAs) and somatic mutations were identified by using whole-genome array-comparative genomic hybridization (aCGH) and targeted next-generation sequencing (tNGS) of 166 to 232 genes (V13 and V14 gene panels are described in the (**Table S1**) as previously described(17,23). tNGS was done using the 2x150-bp paired-end technology on the IlluminaMiSeq or NextSeq500 platforms according to the manufacturer's instructions (Illumina, San Diego, CA, USA). Sequence data were aligned to the human reference genome (UCSC hg19) using Burrows-Wheeler Aligner(24) and processed according to GATK GATK Best Practices recommendations(25). Somatic variant calling was done with Somaticseq software(26). All variants were then annotated for genes and function using ANNOVAR (27). Single nucleotide variants (SNVs) calling was done with Varscan2 version v2.3.8 (28) with a minimal alternate variant frequency and coverage set at 0.02 and 10. Insertions/deletions (indels) calling was done with GATK Haplotype Caller version 3.72 (25) with default parameters and Pindel(29). The variants, *i.e.* SNV and indels were annotated with the Annotate Variation Software ANNOVAR. Known variants found in dbsnp129 and dbsnp137 with a frequency superior to 1% (1000G or ESP6500) were removed. Finally, low frequency SNVs and indels that were suspected to be false positive were systematically inspected with IGV version 2.3.32 (30,31).

To define actionable genetic alterations, we applied the algorithm developed by Perera-Bel et al.(32,33),which matches patient-specificgenomicalterations to treatment options. Thismodel is based upon public knowledge of somatic variants with predictive evidence on drugresponse, including the Gene-Drug-Knowledge-database (GDKD), the Clinical-Interpretation-of-Variants-in-Cancer(CIViC), and the Tumor-Alterations-Relevant-for-Genomics-driven-Therapy (TARGET). The molecular alterations of the actionable genes are classified into a six-level system to rank the associations, according to their evidence, into two axes: strength of clinical evidence (axis 1: treatment approved, 2: treatment in clinical trial, 3: preclinical study) and cancer type (axis A: evidence in the same cancer type and B: evidence in any other cancer type). The highest level is A1, then B1, then A2, then B2, then A3, then B3. In our analysis, we considered only alterations noted as associated with "response" or "sensitivity" to drugs, by excluding those associated with "no response" or "resistance".

Thus in our study, a given mutation was considered actionable in a given patient if 1) classified A1, A2 or B1, B2; 2) the patient had not yet been exposed to the corresponding drug and 3) based on literature data showing

higher response rate of TET2 mutations when treated with hypomethylating agents(34), TET2 was thus considered A2 in our study.

Ex vivo drug sensitivity and resistance profiling (DSRP)

DSRP was done on fresh mononuclear cell samples from refractory/relapsed AML patients as well as peripheral blood mononuclear cells (PBMC). In brief, 20,000 cells per well were arrayed into four, 96-well plates containing a panel of 78small-molecule drugs at graded concentrations (**Table S2**). The panel includes conventional chemotherapeutics and a broad range of targeted oncology compounds. Drug panel plates were created using inhibitors purchased from LC Laboratories and Selleck Chemicals and master stocks were reconstituted in dimethylsulfoxide (DMSO) and stored at−80°C. Every compound was tested in four concentration ranges separated by one log at a constant DMSO level (1%). Master plates containing dilutions of compounds at 100X in DMSO were made and 5µLof these dilutions were plated using a multichannel pipette Eppendorf Xplorer in daughter plates. The latter were then sealed by an aluminum film (Costar 6570) and stored at -80°C. Cells were seeded into assay plates in Roswell Park Memorial Institute (RPMI) 1640 medium supplemented with fetal bovine serum (FBS) (20%), L-glutamine, penicillin–streptomycin, and β-mercaptoethanol(10^{-4} M).After48 h of culture at 37°C in 5% CO_2, cell viability was measured using the CellTiter-Glo luminescent assay (Promega, Charbonières les Bains, France). The data were normalized to negative control wells (DMSO-only) and effective half-maximal concentration values (EC50) were deduced from dose-response curves obtained using GraphPadPrism 6 software (GraphPad Software, Inc.).

Supplemental Tables and Figures

Table S1: Gene panel

Genes in V13 panel of september 2015					Added genes in V14 panel of january 2017	
ABCC9	CSNK1A1	HES1	MEIS1	PTPRT	ANKRD11	OBSCN
ABL1	CTCF	HUWE1	MFSD11	RAD21	ARIH1	OGT
AKT1	CUX1	IDH1	MLL3	RB1	ATG2B	PPM1D
ANKRD26	CXCR4	IDH2	MPL	RIT1	ATRX	PTCH1
APC	DAXX	IKZF1	MYBL2	RUNX1	BCR	PTPN1
ARNTL	DDX3X	IL7R	MYC	SCRIB	CDC25C	RAC1
ASXL1	DDX41	IRF1	MYD88	SETBP1	CDH23	RAC2
ASXL2	DDX54	JAK1	NCSTN	SETD2	CDKN1B	RBBP6
ASXL3	DHX29	JAK2	NF1	SF3B1	CRNKL1	RBL1
ATM	DNMT3A	JAK3	NFE2	SH2B3	CSMD1	RCOR1
BAP1	DOCK2	KDM6A	NFIA	SMC1A	DDX11	RCOR2
BARD1	DOK1	KIF17	NOTCH1	SMC3	DNAH2	RCOR3
BCL11B	DOK2	KIT	NOTCH2	SPIB	DNMT1	ROBO1
BCOR	E2F2	KLF1	NOTCH3	SRSF2	EGLN1	ROBO2
BCORL1	EED	KLHL6	NOTCH4	STAG1	EPOR	RRAS
BMI1	EP300	KMT2A	NPM1	STAG2	FAT1	RRAS2
BRAF	ERG	KMT2A	NRAS	STAT3	FAT4	SIL1
BRCC3	ETNK1	KMT2D	PAX5	STAT5A	FES	SOCS2
CALR	ETNK2	KRAS	PDGFRA	STAT5B	GSKIP	SPI1
CBL	ETS1	LAMB4	PDS5B	SUZ12	HHEX	TET1
CBLB	ETS2	LDB1	PHF6	TAL1	IDH3B	TET3
CDKN2A	ETV6	LEF1	PIAS2	TAL2	JARID2	TRIM10
CDKN2B	EZH2	LMO1	PIK3R2	TCF12	KANSL1	TYK2
CDYL	FBXW7	LMO2	PIM1	TCF3	KANSL2	USP9X
CEBPA	FLT3	LMO3	PIM2	TCF7	KANSL3	WDR5
chr16:820.183-820.277	FOXP1	LUC7L2	PIM3	TET2	KDM3B	ZNF717
chr17:74.732.532 -74.732.630	GATA1	LYL1	PRDX2	TP53	KDM5A	
chr4:153.258.807 -153.259.248	GATA2	MAFK	PRMT5	TRIM33	KDM5C	
chr7:139.102.209 -139.112.272	GATA3	MAML1	PRPF8	U2AF1	MED12	
chr8:144.895.127 -144.895.212	GFI1	MAPK1	PTEN	WHSC1	MSI2	
CLSTN1	GFI1B	MDM2	PTK2B	WT1	NAMPT	
COPA	GNAS	MECOM	PTP4A3	ZMYM3	NIPBL	
CREBBP	GNB1	MEF2C	PTPN11	ZRSR2	NSD1	
CSF3R						

Table S2: Panel of drugs

Molecules	1srt dose (µM)	Classe
	**DNA**	
Cladribine	0.2	
Clofarabine	20	Antimetabolite
Cytarabine	20	
Bleomycine sulfate	50	DNA damage
Nelarabine	50	Purine analogue
Topotecan		Inhibition topoisomerase I
daunorubicin	2	
Etoposide	20	Inhibition topoisomerase II
Idarubicin	0.2	
	**Epigenetic**	
GSK126	50	EZH2 inhibitor
EPZ5676	50	DOTL1 inhibitor
GSK2879552	50	LSD1 inhibitor
JQ1	20	BET bromodomain inhibitor
OTX015	20	
Azacitidine	50	
Decitabine	50	DNA hypo methylating agent
SGI110	50	
SGI-1027	20	
AGI-5198	50	IHD1 inhibitor
Enasidenib (AG-221)	50	IHD2 inhibitor
Entinostat (MS-275)	20	
Vorinostat (SAHA)	20	HDAC inhibitor
Panobinostat	0.02	
	**Apoptosis**	
Navitoclax	2	anti BCL2
Venetoclax	0.02	
Nutlin 3	50	MDM2 inhibitor
idasanutlin	50	p53/MDM2 inhibitor
palbociclib	50	CDK4/6 inhibitor
Seliciclib	50	CDK inhibitor
Olaparib	20	PARP inhibitor
Prima1	20	re activator of mutant p53
Birinapant	2	XIAP cIAP inhibitor
	**Others**	
Dexamethasone	20	immunomodulator
Metformin	24000	AMPK activation. inhibition mTOR
hydroxychloroquine	100	autophagy
Selinexor	2	CRM1 inhibitor
Tretinoin	2	Cells differenciation
Cyclopamine	50	Smo antagonist
Tipifarnib	50	Farnesyl transeferase inhibitor
L-Asparaginase	50	Asparigine depletion
Cyclopamine	50	Smo antagonist
Arsenic	50	Degradation PML/RARA

Molecules	1srt dose (µM)	Classe
	**Microtubule**	
Docetaxel	0.2	Antimitotique
Vincristine	2	
	**Proteasome**	
17-AAG	50	anti heatshock protein
Bortezomib	0.2	Proteasom inhibitor
Carfilzomib	0.2	
Luminespib	0.02	HSP90 inhibitor
	**Small kinases**	
Alisertib		Aurora A inhibitor
VX680	20	Aurora inhibitor
AT9283	20	JAK 2/3 inhibitor
Ruxolitinib	50	Janus kinase 1/2 inhibitor
Trametinib	2	MEK MAPK/Erk Kinase inhibitor
Ponatinib	20	multi target BCR ABL kinase inhibitor
Vemurafenib	50	B-Raf inhibitor
Imatinib	20	v-abl. c-kit. PDGFR inhibitor
Volasertib		Polo like kinase inhibitor
Bosutinib	20	Scr/abl inhibitor
Nilotinib	20	BCR ABL kinase inhibitor
Dasatinib	20	SFK et ABL family inhibitor
	**Receptor tyrosine kinases**	
Afatinib	50	
Erlotinib	20	EGFR family inhibitor
Gefitinib	50	
Masitinib	20	c-kit PDGF inhibitor
Lapatinib	20	EGFR et HER2 inhibitor
Regorafenib	50	multi target BCR ABL kinase inhibitor
SGI-1776	50	PIM. Flt3 inhibitor
Rigosertib	2	Plk1 et PI3K inhibitor
Midostaurin	20	anti FLT3. multi target TK inhibitor
Crenolanib	50	FLT3/ PDGFR inhibitor
Gilteritinib	20	FLT3/AXL inhibitor
Quizartinib	20	Fms like TK3 inhibitor
Sorafenib tosylate	20	multi target FLT3 inhibitor
Sunitinibmalate	20	
	**PI3K pathway**	
Everolimus	50	Mtor inhibitor
Temsirolimus	20	
MK-2206	20	Pan-Ak tinhibitor
BKM 120	20	PAN-PI3K inhibitor
Idelalisib	50	PI3K inhibitor

Table S3: Characteristics of the TTS group

Number inclusion	WHO Classification 2016	ELN at inclusion	Previous line number	Mutations in NGS	DSRP	Correlation between actionnable target and DSRP	Chemogenomic guided treatment	Received Treatment based on	Cycle number	Best objective Response
**1018**	AML MRC	Adverse	2	*DNMT3A.IDH2. PHF6.NF1*	AGI-5198. AGI-6780.dexamethasone. arsenic	Yes	Anti-PI3K/Akt/mTOR with PF-05212384 (PKI-587)	NGS	1	No##
**1020**	AML MRC	Intermediate	1	*SFSRF2.IDH2*	Nelarabine. prima 1	No	AG-221-AML-004 - IDHENTIFY	NGS	5	No #
**1040**	Therapy-related	Intermediate	1	*IDH2*	Resistant	NA	AG-221-AML-004 - IDHENTIFY	NGS	1	No ##
**1002**	AML MRC	Adverse	3	*SF3B1. KMT2C. DNMT3A.TET2*	Dexamethasone.vincristine. rigosertib	Yes (RAS activation via SH3B19 loss)	Dexamethasone + vincristine	DSRP	4	No#
**1004**	AML MRC	Adverse	4	*SF3B1. GATA2*	Cytarabine.midostaurin. tramétinib.tipifarnib. L-asparaginase. OTX015. JQ1	NA	Vincristine + asparaginase	DSRP	1	No##
**1007**	AML MRC	Adverse	3	*NRAS. SRSF2. ASXL1. RUNX1. TET2*	Dasatinib.idelalisib.everolimus. trametinib	Yes	Dasatinib	DSRP	2	No#
**1019**	AML MRC	Adverse	3	*JAK2. RUNX1*	Cladribine.clofarabine. prima1. nutlin3.vidaza	No	Cladribine based conditionning regimen	DSRP	NA	Yes **
**1024**	CMML 2	Adverse	3	*JAK2. ASXL1. U2AF1. TP53. Rad21.NRAS. ETV6*	Aracytine.clofarabine. cladribine.dasatinib.ruxolitinib. MK-2206 2HCl.AT9283. BKM 120. VX680	Yes	Aracytine + dasatinib	DSRP	1	NA
**1025**	AML MRC	Intermediate	2	*GATA2. SRSF2.TET2. SUZ12*	Navitoclax	No	CL1-055746-002 Protocole (Bcl2 inhibitor S055746)	DSRP	6	No ##
**1039**	Therapy-related	Adverse	2	*NRAS. STAG25. SETD2*	Trametinib.cytarabine. L-asparagine.idelalisib	Yes	Idelalisib	DSRP	1	No ##
**1046**	Therapy-related	Intermediate	3	*PTEN.TET2.NOTCH1. CREPPB. IKZF1.NRAS. BCOR2.TRIM33. CTCF*	cyclopamine. AT9283. VX680.azacitidine.topotecan	Yes	Topotecan + aracytine	DSRP	1	No ##
**1050**	AML with mutated NPM1	Favorable	5	*TET2. NPM1.NRAS. PTCH1. NPE2.PTPN11. FOP1. TCF12*	Idarubicine.daunorubicine. clofafarabine.cyclopamine. azacitidine	Yes	Azacitidine	DSRP	5	Yes **
**1017**	AML with mutated NPM1	Favorable	1	*DNMT3A. TET2. NPM1.FLT3*	Sorafenib.dasatinib.temsirolimus.everolimus.bosutinib	Yes	Azacitidine + sorafenib	BOTH	20	Yes**
**1021**	AML with mutated NPM1	Favorable	2	*WT1. NPM1.FLT3*	Metformine.sorafenib et anti BCL2	Yes	Azacitidine + sorafenib	BOTH	12	Yes **
**1029**	AML MRC	Adverse	1	*TP53. DNMT3A*	Anti MEK. MK-2206. BKM 120. prima1.panobinostat.	Yes (KRAS amplication)	Subcutaneousaracytine + cobimetinib	BOTH	1	No ##
**1033**	AML with t(6;9)(p23;q34.1)	Adverse	3	*FLT3. ATM. EZH2*	Nutlin3.sorafenib	Yes	Azacitidine + sorafenib	BOTH	3	Yes *
**1041**	AML MRC	Adverse	1	*TP53.JAK2.NRAS. TET2. EZH2.NF1.PTPN11.SF3B1. DNMT3A. PHF6*	Trametinib. L-asparaginase. vincristine. prima1. AT8283.erlotinib.vorinostat	Yes	Asparaginase + vincristine+ ruxolitinib	BOTH	1	No #

* did not meet working group criteria for response. ** CR. # decrease peripheral blast but progressed after. ## progressed. NA no applicable

Table S4: Correlations between actionable mutations found in the patients and sensitivity to drugs in DRSP.

Actionable Genes	Mutated patients	Potential therapies (level of evidence)	References (PMID)	Patient sensitive in DRSP (Z score < -0.5)	Mean EC50 mutated patients	Mean EC50 WT patients	False Discovery Rate
TP53	11	P53 reactivator (A2)	18341636	7 patients (64%)	0,2	0,28	1
		Decitabine (A2)	27959731	0 patient	0,23	0,26	1
		HSP90i (A3)	26009011	4 patients (36%)	0,51	0,32	1
		Pramlintide (B3)	25409149	-	-	-	-
SRSF2	9	Spliceosome inhibitors (A3)	27135740	-	-	-	-
NRAS	7	MEKi (A2/B2)	26990290, 23414587, 18390968, 23515407, 22985491	3 patients (43%)	0,63	0,97	0,95
		HSP90i (B3)	23538902	6 patients (86%)	0,04	0,43	*0,01*
PTPN11	7	SHP2/PTPN11i (A3)	30457860	-	-	-	-
		MEKi (A3/B3)	26365186, 17053061	2 patients (28%)	0,82	0,94	1
NF1	6	MEKi (B2)	NCT01362803	1 patient (17%)	0,82	0,94	1
		Imatinib (B3)	23099009	0 patient	1	0,95	0,44
		MTORi (B3)	18483311	2 patients (33%)	0,24	0,32	0,91
		BETi (B3)	24373973	6 patients (100%)	0,08	0,43	*0,02*
BCOR	5	enzastaurin (B3)	27397505	-	-	-	-
EZH2	5	EZH2i (B2)	27397505	-	-	-	-
		EZH2i + HDACi or + decitabine (A3)	19638619	-	-	-	-
IDH2	5	IDH2i (A1)	28588019	1 patient (20%)	0,8	0,9	1
SF3B1	5	Spliceosome inhibitors (A3/B3)	30054334, 25424858	-	-	-	-
JAK2	4	JAK2i (B1/A2)	27211272	0 patient	0,96	0,91	1
U2AF1	4	FLT3i (A3)	27397505	1 patient (25%)	0,49	0,45	1
		Spliceosome inhibitors (A3)	30054334	-	-	-	-
FLT3	3	FLT3i (A1)	30721452	2 patients (67%)	0,26	0,45	1
NOTCH1	3	NOTCH1i(B2)	27870570	-	-	-	-
		Notch reactivator (A3)	27211848	-	-	-	-
STAG2	3	PARP i (B3)	24356817	-	-	-	-
DNMT3A	2	Decitabine (A2)	22124213	0 patient	0,34	0,23	1
APC	2	Tankyrase inhibitors, WNTi (B3)	22440753	-	-	-	-
CBL	2	JAKi (B3)	23696637	0 patient	-	-	-
		Dasatinib (B3)	23696637	1 patient (50%)	-	-	-
DDX41	2	Lenalidomide (A3)	25920683	0 patient	-	-	-
KRAS	2	MEKi (A2)	26990290	0 patient	-	-	-
PTCH1	2	Vismodegib (B2)	21300762, 22670903	1 patient (100%)	-	-	-
ATM	1	ATRi, PARPi, cisplatine (B2)	26510020, 26238431	-	-	-	-
		Temozolomide (B3)	23960094	-	-	-	-
CALR	1	JAK2i (B2)	-	0 patient	-	-	-
CUX1	1	PI3K AKTi (A3/B3)	24316979, 25190083	0 patient	-	-	-
FAT1	1	BETi (B3)	26365186	0 patient	-	-	-
IDH1	1	IDH1i(A1)	29860938	0 patient	-	-	-

Table S5: Significant correlations between molecular profile and sensitivity to drugs

Associations	Number of mutated patients	Number of WT patients	Mutated EC50 median	WT EC50 median	Fold change	False Discovery Rate	Drug classes
NRAS_MUT_AT9283 MO	5	31	0,08	0,64	-0,55	2,00E-06	JAK 2/3 inhibitor , Aurora A/B, Abl(T315I)
NRAS_MUT_MK2206 MO	5	32	0,04	0,41	-0,37	2,64E-05	Pan-Akt inhibitor
RUNX1_MUT_Bleomycine MO	5	29	0,25	0,79	-0,54	2,64E-05	DNA damage
NRAS_MUT_VX680 MO	5	29	0,14	0,79	-0,65	5,82E-05	Aurora inhibitor
RUNX1_MUT_Idarubicine MO	6	31	0,07	0,32	-0,25	2,26E-03	Inhibition topoisomerase II
EZH2_MUT_Cladribine MO	4	32	1,00	0,61	0,38	3,15E-03	Antimetabolite
JAK2_MUT_Entinostat SG	4	38	0,07	0,47	-0,40	3,15E-03	HDACi
NRAS_MUT_Topotecan SG	7	35	0,09	0,47	-0,38	3,31E-03	Inhibition topoisomerase I
NRAS_MUT_MK2206 SG	7	36	0,09	0,44	-0,36	4,12E-03	Pan-Akt inhibitor
NRAS_MUT_17AAG MO	5	30	0,04	0,43	-0,39	6,02E-03	**heat shock protein inhibitor**
TP53_MUT_Bleomycine MO	8	26	1,00	0,62	0,38	9,11E-03	DNA damage
NRAS_MUT_Ponatinib MO	5	32	0,02	0,23	-0,21	9,80E-03	multi target BCR ABL kinase inhibitor
NRAS_MUT_Selinexor MO	4	14	0,06	0,70	-0,64	9,80E-03	CRM1 inhibitor
NRAS_MUT_Venetoclax SG	5	18	0,40	0,91	-0,51	9,80E-03	anti BCL2
TP53_MUT_Bleomycine SG	10	32	1,00	0,72	0,28	9,80E-03	DNA damage
NRAS_MUT_OTX015 MO	5	32	0,04	0,35	-0,32	1,03E-02	BET bromodomain inhibitor
RUNX1_MUT_Clofarabine MO	5	31	0,10	0,68	-0,58	1,08E-02	Antimetabolite
TP53_MUT_Nutlin 3 SG	10	32	0,90	0,52	0,37	1,35E-02	p53/ MDM2 inhibitor
NRAS_MUT_Hydroxychloroquine SG	7	34	0,24	0,51	-0,27	1,63E-02	autophagy
JAK2_MUT_Topotecan SG	4	38	0,10	0,44	-0,34	1,70E-02	Inhibition topoisomerase I
NRAS_MUT_Afatinib MO	5	30	0,17	0,44	-0,26	1,70E-02	EGFR/HER2 inhibitor
DNMT3A_MUT_Selinexor MO	4	14	0,10	0,69	-0,58	1,73E-02	CRM1 inhibitor
EZH2_MUT_AraC SG	5	38	1,00	0,77	0,23	1,73E-02	Antimetabolite
NF1_MUT_AraC SG	5	38	1,00	0,77	0,22	1,73E-02	Antimetabolite
NF1_MUT_Bleomycine SG	5	37	0,99	0,76	0,24	1,73E-02	DNA damage
NF1_MUT_JQ1 MO	5	31	0,08	0,43	-0,36	1,73E-02	BET bromodomain inhibitor
NRAS_MUT_Gilteritinib MO	4	14	0,08	0,61	-0,54	1,73E-02	FLT3/AXL inhibitor
NRAS_MUT_Ponatinib SG	7	36	0,05	0,27	-0,22	1,73E-02	multi target BCR ABL kinase inhibitor
NRAS_MUT_Regorafenib MO	5	30	0,16	0,48	-0,32	1,73E-02	VEGFR, Kit, RET Raf-1 inhibitor
NRAS_MUT_Sorafenib SG	7	35	0,19	0,49	-0,30	1,73E-02	Multikinase inhibitor (B-Raf, Raf-1, VEGFR)
NRAS_MUT_Topotecan MO	5	30	0,05	0,37	-0,31	1,73E-02	Inhibition topoisomerase I
NRAS_MUT_Volasertib SG	7	36	0,11	0,42	-0,31	1,73E-02	Polo like kinase inhibitor
PTPN11_MUT_JQ1 MO	6	30	0,07	0,45	-0,37	1,73E-02	BET bromodomain inhibitor
PTPN11_MUT_Panobinostat MO	4	14	0,23	0,80	-0,57	1,73E-02	HDACi
SF3B1_MUT_Lapatinib MO	4	31	0,84	0,52	0,32	1,73E-02	EGFR et HER2 inhibitor
TP53_MUT_Idarubicine SG	10	33	0,70	0,29	0,41	1,73E-02	Inhibition topoisomerase II
TP53_MUT_Nutlin 3 MO	9	26	0,90	0,52	0,39	1,73E-02	p53/ MDM2 inhibitor
JAK2_MUT_JQ1 SG	4	39	0,12	0,54	-0,42	2,25E-02	BET bromodomain inhibitor
NRAS_MUT_JQ1 MO	5	31	0,06	0,44	-0,37	2,25E-02	BET bromodomain inhibitor
NRAS_MUT_Volasertib MO	5	32	0,08	0,32	-0,24	2,25E-02	Polo like kinase inhibitor
PTPN11_MUT_Entinostat MO	6	28	0,04	0,37	-0,32	2,25E-02	HDACi
NRAS_MUT_Daunorubicine SG	7	36	0,04	0,28	-0,24	2,48E-02	Inhibition topoisomerase II
NRAS_MUT_Sorafenib MO	5	30	0,22	0,50	-0,28	2,69E-02	Multikinase inhibitor (B-Raf, Raf-1, VEGFR)
NRAS_MUT_Azacitidine MO	5	30	0,25	0,66	-0,40	2,70E-02	DNA methylation inhibitor
RUNX1_MUT_Prima1 MO	6	30	0,09	0,29	-0,20	2,72E-02	re activator of mutant p53
EZH2_MUT_AraC MO	4	33	1,00	0,77	0,23	2,73E-02	Antimetabolite
SF3B1_MUT_Entinostat MO	4	30	0,05	0,34	-0,30	3,28E-02	HDACi
NRAS_MUT_Regorafenib SG	7	35	0,16	0,45	-0,29	3,46E-02	VEGFR, Kit, RET Raf-1 inhibitor
NRAS_MUT_Bortezomib SG	7	36	0,04	0,26	-0,23	3,46E-02	proteasom inhibitor
TP53_MUT_Nelarabine SG	10	32	1,00	0,80	0,20	3,70E-02	Purine analog
NRAS_MUT_Daunorubicine MO	5	31	0,03	0,25	-0,22	4,87E-02	Inhibition topoisomerase II
NRAS_MUT_OTX015 SG	7	36	0,10	0,48	-0,37	4,87E-02	BET bromodomain inhibitor

Figure S1 : Oncoprint representing all the mutations presented by the patients, the type of mutations and the functional category they belong to.

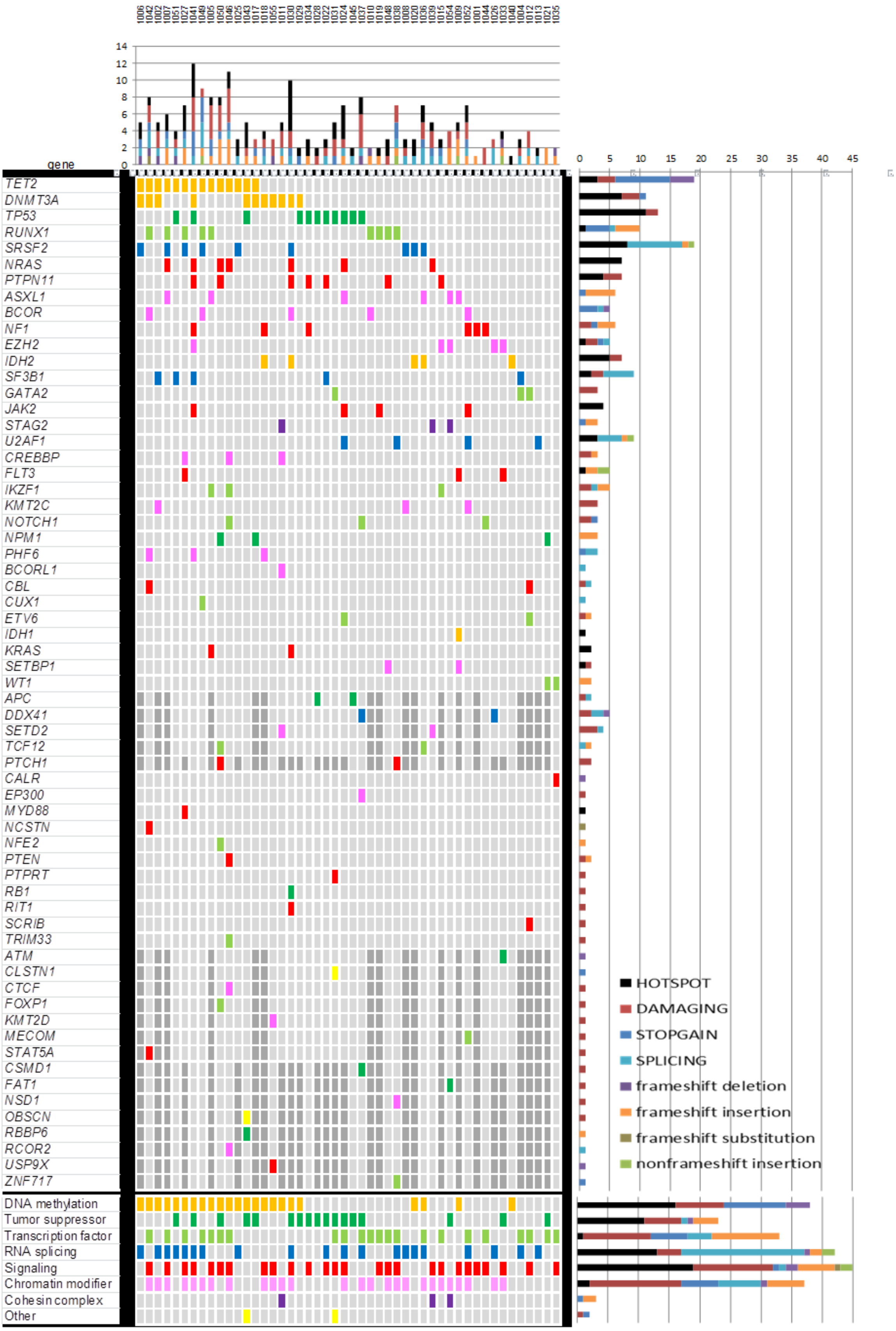

Figure S2: Difference in terms of level of sensitivity of each patient between blood and bone marrow. We observed a noteworthy difference in patient 1022 (more sensitive in blood) and patients 1029 and 1034 (more sensitive in bone marrow), not explained by a difference in blast percentage (indicated at the top of the bars)

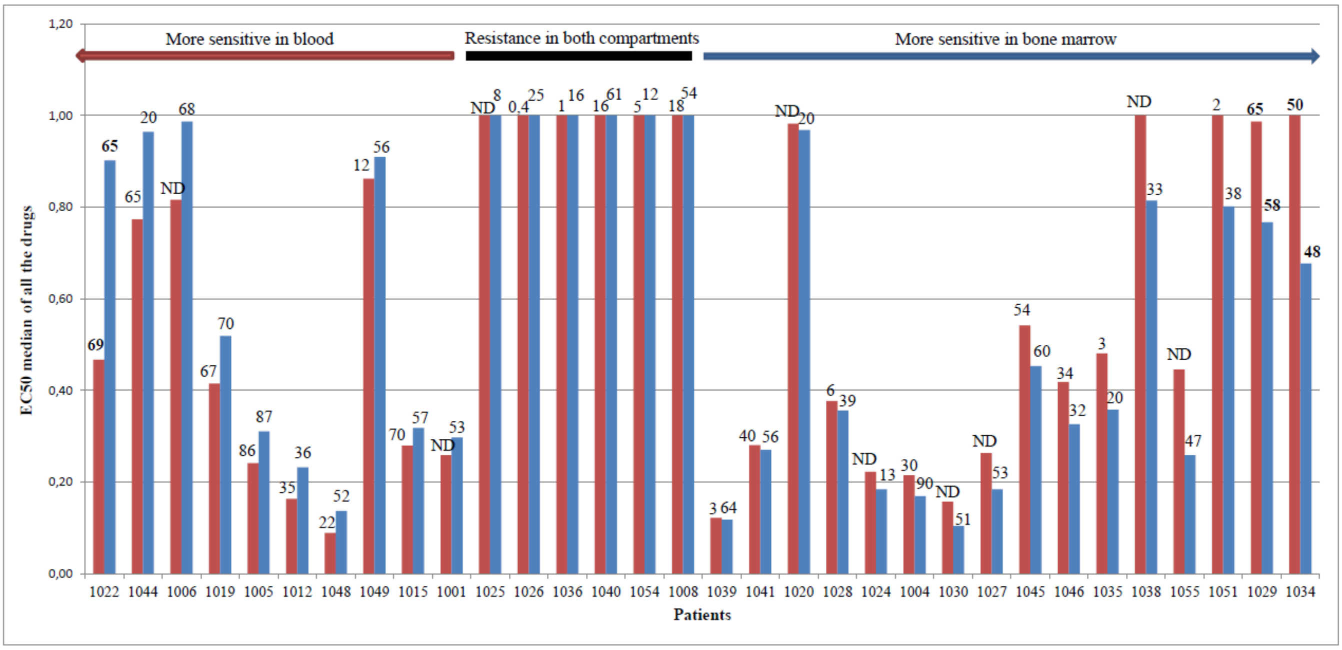

Figure S3 : Representation of all the drugs proposed in MRB based on results of DRSP. Legend: i: inhibitor; TKR: Tyrosine kinase inhibitor; HMA: hypomethylating agent; BRDi: bromodomaine inhibitor; HDACi: histone deacetylase inhibitor; FTi: farnesyl transferase inhibitor.

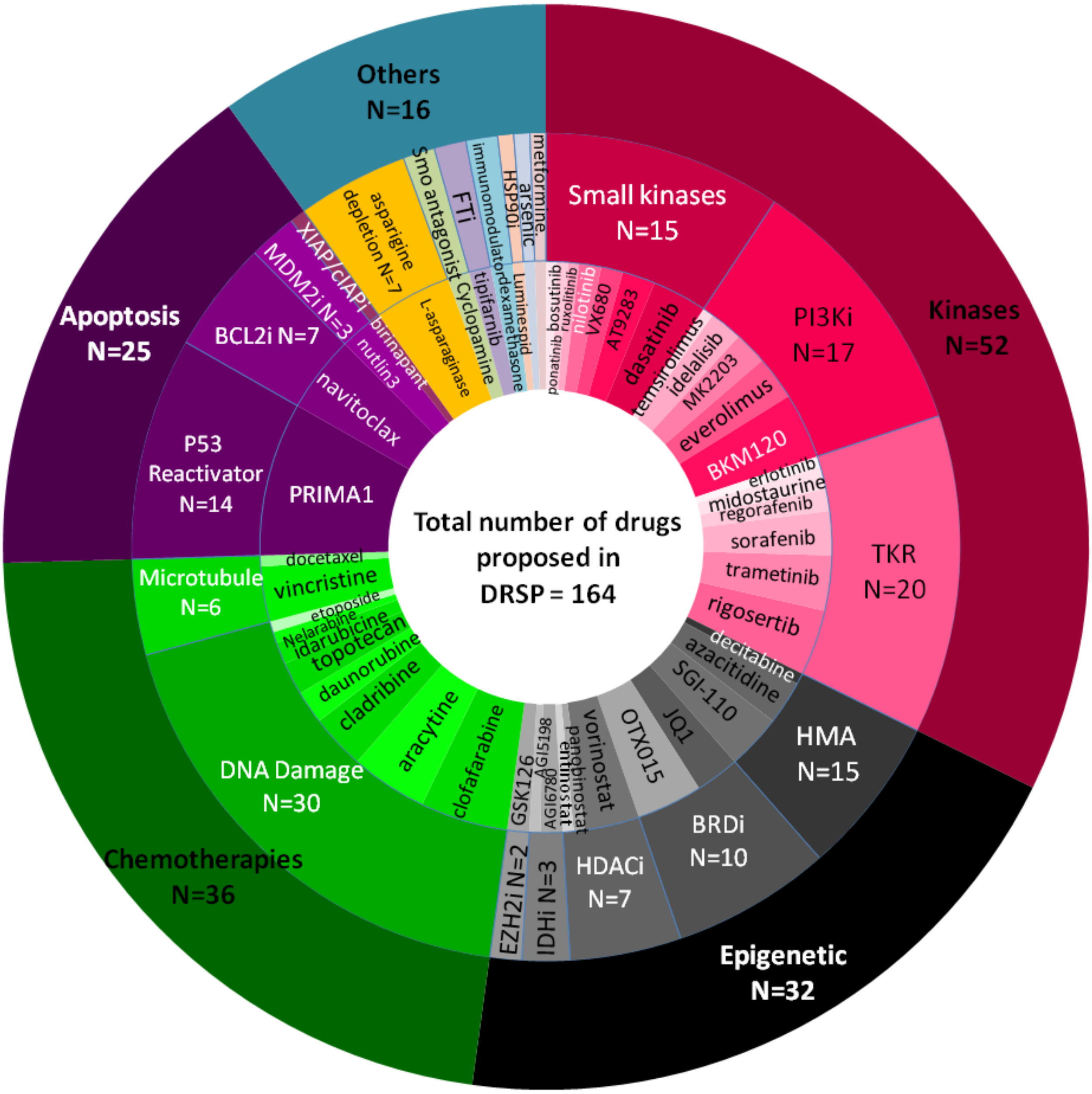

Figure S4 : Global level of sensitivity of the cohort. Each point represents one patient by the median of all the normalized EC50s of the drug panelwith profiles rather « resistant » in red and «sensitive» profiles in green.

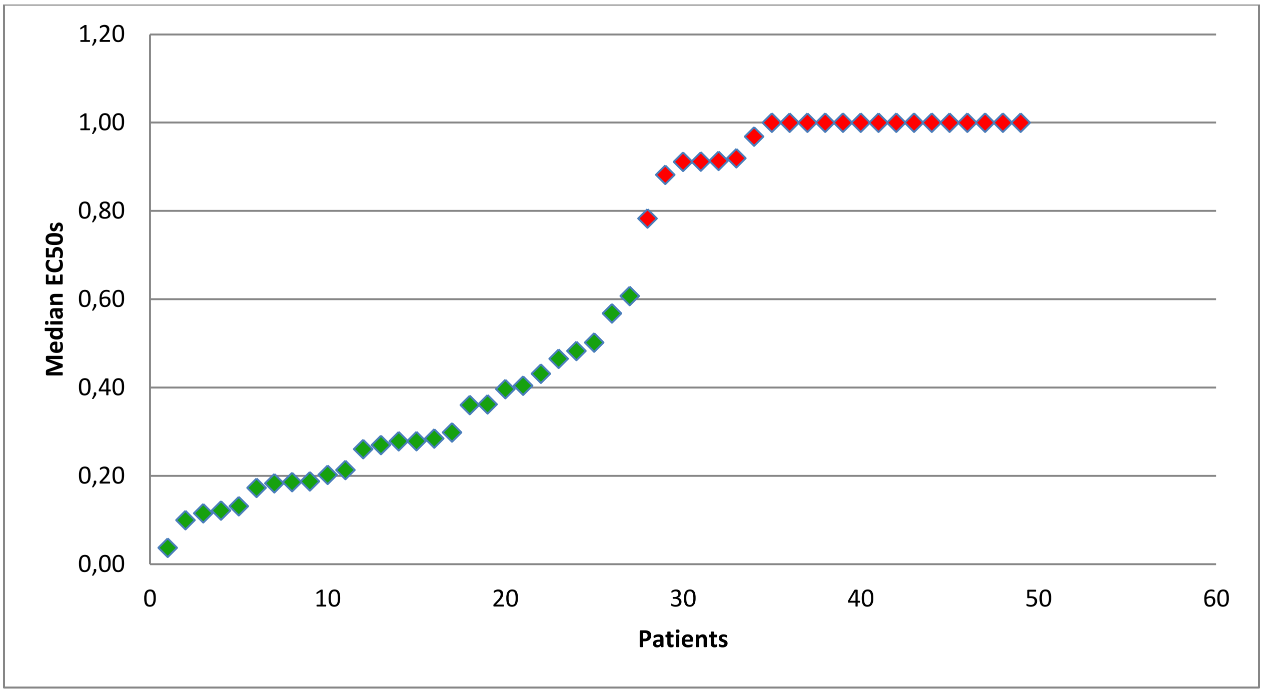

Figure S5 : Clustering representing all the normalized EC50 for each drug and each patient with the color green defining sensitivity and red resistance. Grey means that the drug was not evaluable for the patient.

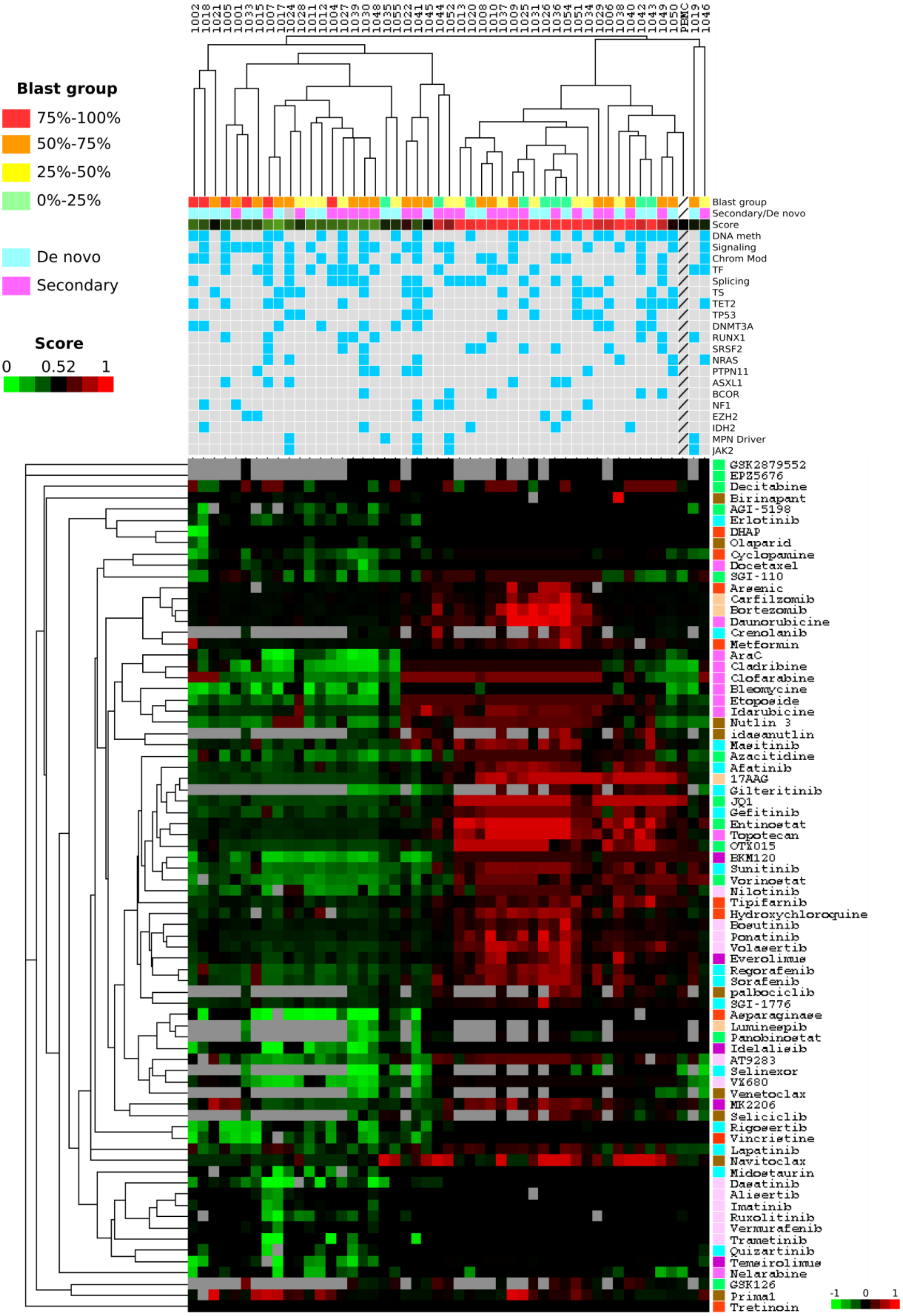

Figure S6 : Boxplot representing the median level of sensitivity in the group having at least one signaling mutation versus the group without.

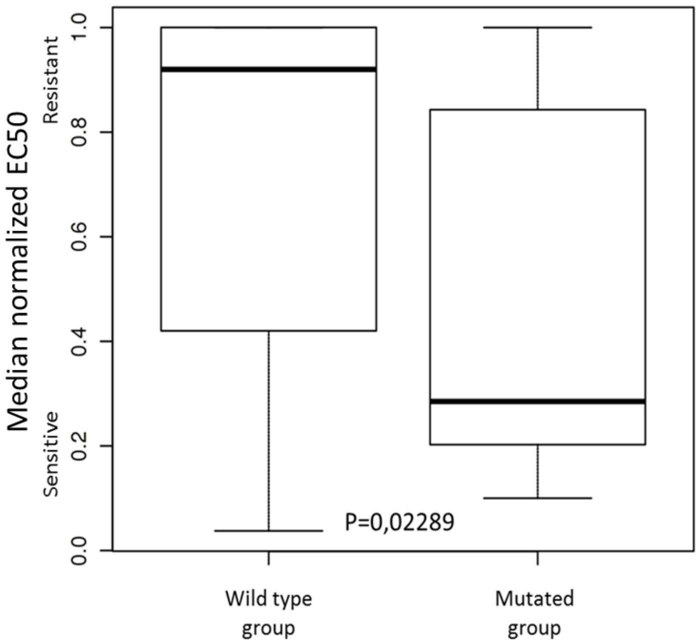

Figure S7: Boxplot representing the median level of sensitivityaccording to the percentage of blasts.

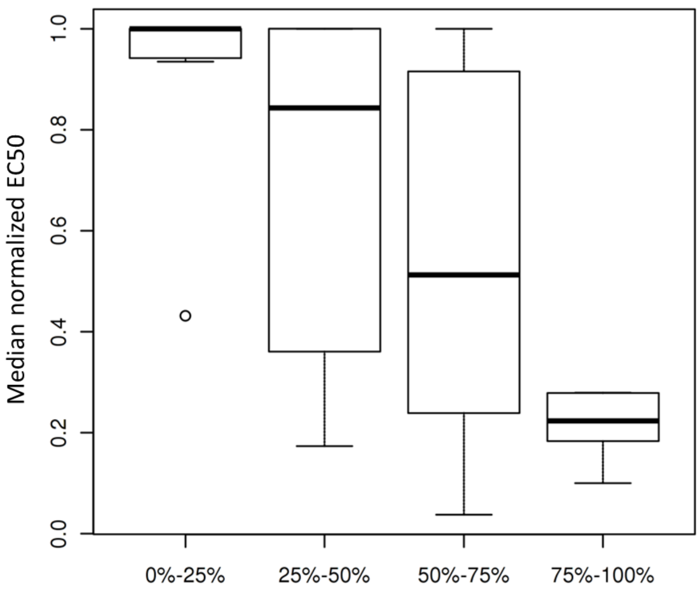

Figure S8 : Boxplot representing the rank of the drugs according to the median of all normalized EC50s of every patient's sample for each drug representing the global level of efficiency of each drug.

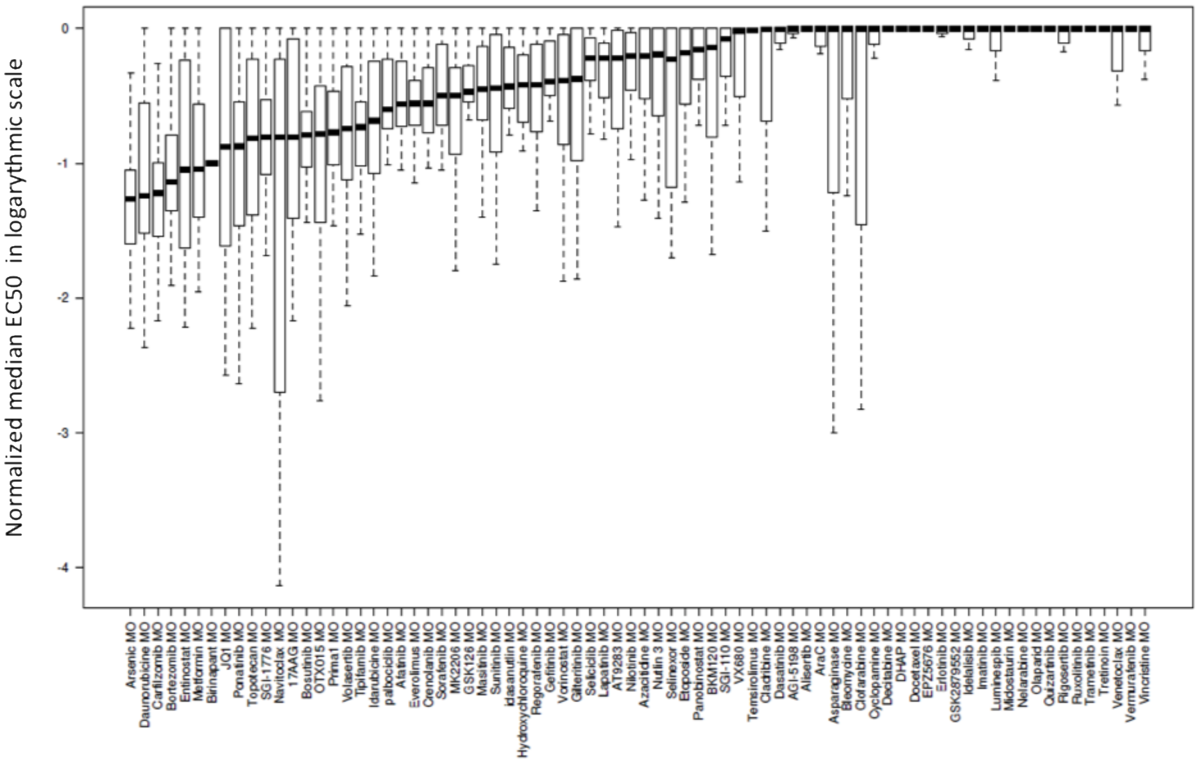

Figure S9 : Boxplot representing the rank of the drugs according to the median of all normalized EC50s of every patient's sample for each drugnormalized by the PBMC EC50 median reflecting the drug specificity of action between leukemic and normal cells.

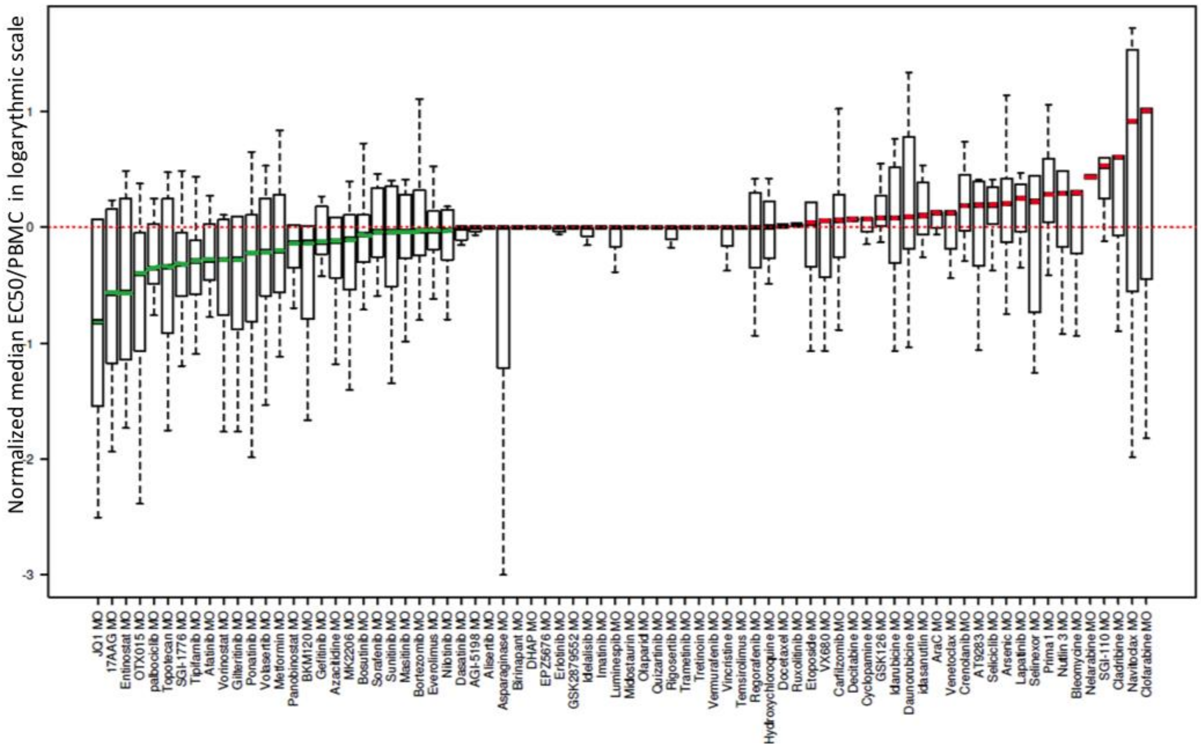

Figure S10: Boxplot representing correlations between drug sensitivity and mutational status

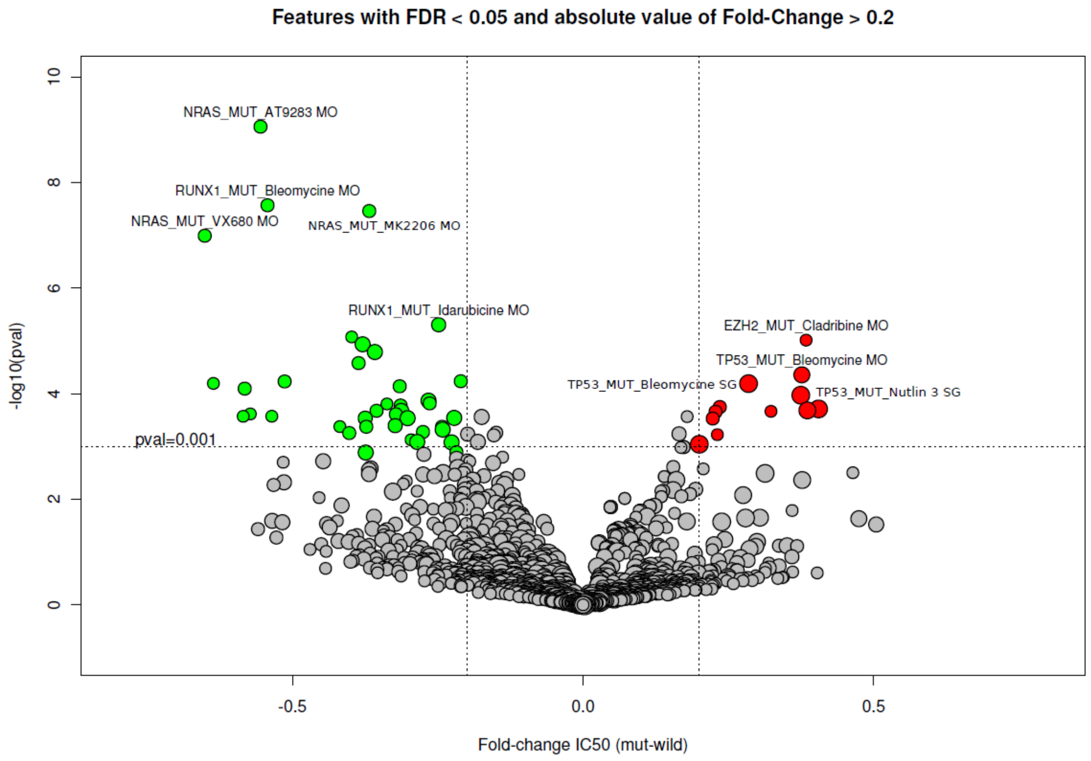

Supplement: Supplementary file 1 — Supplemental material [file 41408_2020_330_MOESM1_ESM.pdf]
